# Temporal niche partitioning among sympatric wild and domestic ungulates between warm and cold seasons

**DOI:** 10.1038/s41598-024-61463-y

**Published:** 2024-05-08

**Authors:** Jian-Feng Wang, Kai Xu, Song Yao, Tong Liu, Bo Yu, Xiao-Qun Huang, Zhi-Shu Xiao, Dong-Po Xia

**Affiliations:** 1 School of Life Sciences, Anhui University, Hefei, 230601 Anhui China; 2 International Collaborative Research Center for Huangshan Biodiversity and Tibetan Macaque Behavioral Ecology, Hefei, 230601 China; 3 State Key Laboratory of Integrated Management of Pest Insects and Rodents, Institute of Zoology, Chinese Academy of Sciences, Beijing, 100101 China; 4 University of Chinese Academy of Sciences, Beijing, 101407 China; 5 Neixiang Management Bureau of Baotianman National Nature Reserve, Neixiang, 474350 Henan China

**Keywords:** Zoology, Ecology

## Abstract

The coexistence of sympatric species with similar ecological niches has been a central issue in ecology. Clarifying the daily activity patterns of sympatric wild ungulates can help understand their temporal niche differentiation and the mechanisms of coexistence, providing information for their conservation. The Baotianman National Nature Reserve in northern China is rich in wild ungulates, but little is known about the daily activity patterns of wild ungulates in the area, making it difficult to develop effective conservation strategies. We studied five representative wild ungulates (i.e. forest musk deer, Chinese goral, Reeve’s muntjac, Siberian roe deer, and wild boar) of the region using camera-trapping data, focusing on the seasonal daily activity patterns and effects of seasonal grazing of domestic sheep, to reveal their coexistence based on temporal ecological niche differentiation. Comparative analyses of the seasonal daily activity showed that forest musk deer exhibited a single-peak activity in the warm season. Other ungulates exhibited multipeak activity. All five ungulates differed significantly in daily activity patterns. Notably, wild boar and Reeve’s muntjac showed high overlap coefficients between the cold and warm seasons. In both cold and warm seasons, the five wild ungulates and domestic sheep displayed low overlap in their daily activity rhythms potentially indicating temporal ecological niche differentiation. The results suggest that temporal isolation might be a strategy for wild ungulates to avoid domestic sheep and reduce interspecific competition, and that temporal ecological niche differentiation potentially promoted the coexistence among the studied sympatric ungulates. This understanding may provide new insights for the development of targeted conservation strategies.

## Introduction

Ecological theory suggests niche differentiation is key to understand species coexistence^1,2^. Effective niche partitioning enhances biodiversity and stabilizes ecosystem functions and community structures^3^. Research showed spatial niche differentiation significantly impacts species coexistence, and differences in habitat selection and use reduce spatial overlap of competing species^4–6^. Moreover, some animals can change their spatial range when interspecific competition becomes increasingly fierce so that they can coexist with competing species in the same landscape through spatial isolation^7,8^. However, many competing species with similar diets, such as ungulates, tend to overlap in terms of space use^9^. With anthropogenic land-use changes and invasions of livestock^10^, competition for space and resources among wild ungulates has been intensifying^11–13^. Habitat reduction due to human activities has further intensified the intensity of competition, making the struggle for the survival of these species more challenging. In this context, temporal ecological niche differentiation may become a major mechanism for the coexistence of wild ungulates^14^.

Daily activity patterns refer to the variations in an animal's activity levels throughout 24 h, influenced primarily by light intensity changes, these patterns stem from the animal's internal physiological mechanisms and natural adaptations to their environment^15–17^. Besides, increasing evidence indicates that the daily activity patterns are also regulated by external factors, such as environmental temperature^18^, rainfall^19^, predator presence^20^, interspecific relationships^15,21^, and human activities^22^. In particular, the change of seasons varies not only in the duration and intensity of light, but also in temperature, precipitation, and even the frequency of human activity. Therefore, seasonal variation is a critical influence on daily activity patterns, especially apparent in wild mammals^23,24^. Moroccan dromedary camels (*Camelus dromedarius*) showed increased nocturnal activity during rainfall to take advantage of lower temperatures and better food resources^25^. In the Apennines of central Italy, European roe deer (*Capreolus capreolus*) increase nocturnal activity in summer to regulate body temperature and synchronize reproduction^17^. Wild boar increase their daytime activity in summer to access abundant food in the Schaalsee UNESCO Biosphere Reserve of northern Germany^26^. Seasonal variation in daily activity patterns may result in changes in temporal ecological niche allocation patterns for sympatric species. For example, chamois (*Rupicapra rupicapra*) tended to be more active during the day when red deer (*Cervus elaphus*) were absent, but showed contrasting patterns of activity across seasons, causing changes in temporal overlap coefficients with red deer^27^. In Mediterranean forests in Italy, the temporal overlap among three ungulates (European roe deer *Capreolus capreolus,* fallow deer *Dama dama,* and wild boar *Sus scrofa*) reached the highest values in summer due to extreme drought and food resources^21^.

In addition, free-range livestock, as non-native ungulates, have entered into competition with native ungulate species for resources within natural ecosystems^22,28^. As competing species with the same trophic level and similar diet structure, based on limited resources, the presence of livestock, in particular, could have a significant impact on sympatric herbivorous ungulates through competitive exclusion^29,30^, resulting the changes in the daily activity patterns of wild ungulates^31^. Moreover, not only competition for resources but also habitat alterations such as fences and other infrastructure lead to strong effects on wild ungulates^32^. In China's Northeast Tiger Leopard National Park, the presence of free-ranging cattle has reduced daytime activities of wild boar and sika deer^31^. Not only does this exacerbate resource competition among herbivores^13,31^, it may also increase their risk of predation^33^. As a result, livestock grazing should be considered an important driver of alteration in activity patterns in wild ungulates^34–36^. Therefore, it is crucial to better understand the daily activity patterns of ungulates, their temporal niche partitioning, and how these factors contribute to their coexistence with humans and domestic animals. However, the patterns of response of different ungulate species to livestock activities, how the temporal ecological niche allocation of ungulates varies across grazing landscapes, and the mechanisms of coexistence between wild ungulates and livestock remain unclear.

The Baotianman Mountains in northern China, designated as a World Biosphere Reserve, play a critical role in the conservation of local biodiversity^37^. This region is home to five native ungulate species: the forest musk deer (*Moschus berezovskii*), Chinese goral (*Naemorhedus griseus*), Reeve's muntjac (*Muntiacus reevesi*), Siberian roe deer (*Capreolus pygargus*), and wild boar^38,39^. Among them, forest musk deer and Chinese goral are only found in a few countries in Asia and are classified as EN and VU species on the IUCN Red List, respectively^40,41^. While Reeve's muntjac, Siberian roe deer, and wild boar are widespread species (LC on IUCN Red List), their role in ecosystems and their sensitivity to environmental change makes them species of high importance for conservation^42–44^. The activity patterns and temporal ecological niche partitioning of these ungulates within China have been the hotspot and core of research and conservation. For example, studies have shown that the daily activity patterns of forest musk in Sichuan vary accordingly with the seasons to search for food at comfortable temperatures^6^; in the presence of Chinese serow, Chinese goral has been shown to increase diurnal activity intensity to gain access to sufficient resources in the exploitation competition^45^; Reeve's muntjac and black muntjac (*Muntiacus crinifrons*) reduced temporal overlap and strengthened temporal niche differentiation under resource-poor conditions to coexist^46^. Thus, temporal niche partitioning is an important coexistence strategy for competing species in resource- or space-constrained situations, and differences in activity patterns of species and assessment of the extent of temporal overlap among them provide important information for understanding the mechanisms of sympatric species interactions and coexistence. However, temporal activity patterns and temporal ecological niche partitioning of ungulates in the Baotianman Mountains are still unclear. In particular, top predators such as leopards (*Panthera pardus*), which were historically present in the region, appear to have disappeared^47^, and there is a large number of free-ranging sheep in the region, even within the protected area^47^. The absence of predators, but also an increase in domestic sheep populations, may cause wild ungulates to adjust their daily activity rhythms to avoid competition with livestock.

Based on the survey data (October 2015 to June 2017, totaling 635 days) from camera-trapping, our study compared the daily activity patterns and seasonal differences between warm and cold seasons of five sympatric wild ungulates and free-ranging domestic sheep in the Baotianman area and explored the temporal ecological niche differentiation patterns of ungulates in the warm and cold seasons and the coexistence mechanisms with domestic animals. We predicted that (1) there would be differences in daily activity patterns among ungulate species, and that temporal niche partitioning would be conducive to their sympatric coexistence. And predicted that (2) differences in daily activity patterns among ungulates would be more pronounced in the cold season due to resource scarcity, i.e., species pairs would have lower temporal overlap coefficients in the cold season than in the warm season.

## Materials and methods

### Study area

**Figure 1:**
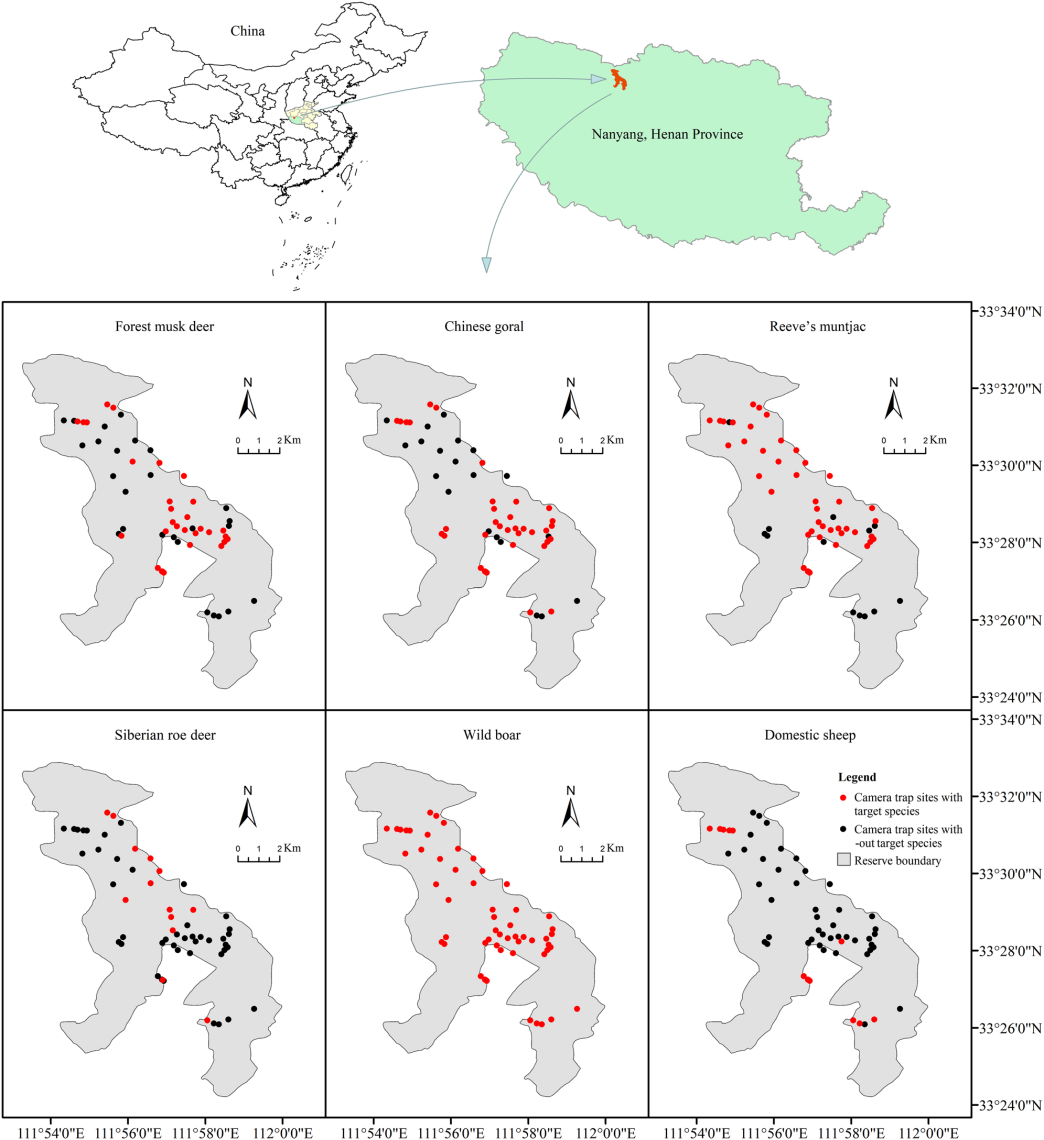
Spatial patterns of animal species detection in the study area captured by cameras in Baotianman National Nature Reserve, Neixiang, from April 2015- June 2017. Red dots represent camera location sites, in which the specified ungulate species was observed, black dots indicate sites in which the species was not observed.

Our study was conducted in the Neixiang Baotianman National Nature Reserve, Henan Province, China (111° 53′–112° E, 33° 25′–33° 33′ N) (Fig. 1). This 9304 km^2^ reserve ranges from 500 to 1950 m above sea level^39^. The average annual temperature is 15.1 °C, with annual precipitation averaging 886 mm. The reserve, predominantly covered by warm temperate deciduous broad-leaved and mixed coniferous-broad-leaved forests, lies in the transitional zone between the warm temperate and north subtropical zones, exhibiting marked seasonal variations (monsoon continental climate, Cwa)^48,49^. Baotianman area is divided into distinct cold and warm seasons throughout the year. The cold season is colder and drier (October–March of next year, the monthly average was 2.4 °C temperature and 21.1 mm precipitation), while the warm season is warmer and wetter (April–September, the monthly average was 16.4 °C temperature and 127.8 mm precipitation)^39,48^. High densities of domestic sheep are the main human-led disturbance for wild ungulates in the reserve. And the herds of domestic sheep in the region are often guarded by shepherds, but are rarely accompanied by dogs (probably because of the lack of large carnivores). In addition, surveys have monitored humans (including rangers and tourists) and a small number of other human-associated species (Supplementary Table S1), but do not pose a threat such as mortality or resource competition to wild ungulates.

### Infrared camera-trapping

As a part of the monitoring of terrestrial mammals and birds in the region^38^, we conducted infrared camera-trapping from April 2015 to June 2017 in Baotianman Nature Reserve. Based on animal footprints and accessibility of the terrain, we set up 62 camera traps (Bestguarder SG-990 V, Nighthawk^47,50^, see Supplementary Table S2 online for specifications) at different sites above sea level from 538 to 1709 m to investigate medium and large mammals (Fig. 1). Generally, we fixed cameras on the trees at a height of 50–80 cm, facing animal tracks, water sources, natural salt lick sites, or resting sites^16,51^. Camera sites were spaced at least 300 m apart from each other. Additionally, no baits were used in camera sites to capture the natural wildlife behavior^1,52^. The shooting mode of the camera trap was set to "Photo + video", which meant three photos and a 30 s video would automatically taken when an animal was detected by the camera. The induction interval for detection was 1 s.

### Kernel density estimation

The images captured by camera-trapping were uploaded to the China Camera Data Network for Wildlife Diversity Monitoring (http://www.gscloud.cn), and each photo of an animal was identified to a species by trained experts. Referring to the method of O'Brien et al.^53^, we defined an independent event as consecutive photographs of individuals of the same species taken more than 0.5 h apart. We compiled independent events for five wild ungulates and domestic sheep and recorded the time and date based on the first photo of the independent event.

We collated the independent events of the wild ungulates and domestic sheep during the cold seasons (that is, from April to September each year during the survey period) and warm seasons (that is, from October to March each year during the survey period), and plotted diurnal active kernel density curves for each species in both the cold and warm seasons, respectively. The recorded clock time of species does not have any biological meaning^54^, so we converted the clock time of independent events to solar time according to the sunrise and sunset time each day at the coordinates of the center of the reserve, and all the solar times were also regarded as a 24-h cycle. To test for activity differences, we generated a null distribution of overlap indices using data sampled randomly with replacement from the combined data and used a randomization test to define an empirical probability that two sets of circular observations come from the same distribution. p < 0.05 indicated that the activity data between the two species were significantly different^55^.

We calculated temporal overlap coefficients (Δ) for the six ungulate species separately for the cold and warm seasons. Additionally, we compared Δ between pairs of ungulate species in the cold and warm seasons, respectively, to explore competitive interactions among them^21^. The Δ was evaluated as the area of overlap under the two activity curves. Thus, Δ represents the extent of overlap in the ecological niche, with values of Δ ranging from 0 to 1. A Δ value of 0 indicates no overlap, while a value of 1 indicates complete overlap. Referring to Ridout and Linkie's method^55^, Δ4 was used for large sample sizes (independent events > 50), while Δ1 was used for small sample sizes (independent events < 50)^56^. And 1,000 smooth bootstraps were generated to estimate confidence intervals (CI) for Δ4 and Δ1. We categorized the overlap coefficients into three levels: Δ < 0.50 for low overlap, Δ = 0.50–0.80 for medium overlap, and Δ > 0.80 for high overlap^57^. All analyses of daily activity patterns and temporal overlap coefficients were conducted using the “activity” and “overlap” packages in R version 4.0.3^55,58^, which was considered to be a commonly used and effective method for camera-trapping time-data analysis.

We targeted the seasonal changes of ungulate activity patterns in warm and cold seasons. Meanwhile, considering the widespread and strong grazing pressure by domestic sheep in the Baotianman area and its effect of competition on the activity patterns of ungulates, we compared the daily activity patterns of the five wild ungulates with those of domestic sheep, to explore their temporal niche differentiation and the mechanism of coexistence.

## Results

During the study, a total of 3860 independent events of ungulates were obtained in 29,060 camera days by 62 camera-trapping sites, which included 87 independent events of forest musk deer, 162 of Chinese goral, 794 of Reeve’s muntjac, 106 of Siberian roe deer, 2588 of wild boar and 123 of domestic sheep (Supplementary Table S1).

### Seasonal daily activity pattern

Forest musk deer, Chinese goral, and Reeve’s muntjac exhibited similar daily activity patterns in cold and warm seasons, with no significant differences (p > 0.05) in activity curves and high overlap coefficients (Δ > 0.80). While daily activity patterns of Siberian roe deer and wild boar differed significantly between the cold and warm seasons (p < 0.01), the same is true for domestic sheep (Table 1).

**Table 1:** Overlap coefficients of daily activities of five wild hoofed species and domestic sheep during the cold and warm seasons based on kernel density estimates.

Species	Overlap coefficient	CI	p value
Wild			
Forest musk deer, *Moschus berezovskii*	0.71	0.61–0.80	0.15
Chinese goral, *Naemorhedus griseus*	0.83	0.71–0.92	0.44
Reeve’s muntjac, *Muntiacus reevesi*	0.83	0.73–0.90	0.23
Siberian roe deer, *Capreolus pygargus*	0.86	0.83–0.89	< 0.01
Wild boar, *Sus scrofa*	0.77	0.71–0.83	< 0.01
Domestic			
Sheep, *Ovis aries*	0.55	0.44–0.64	< 0.01

**Figure 2:**
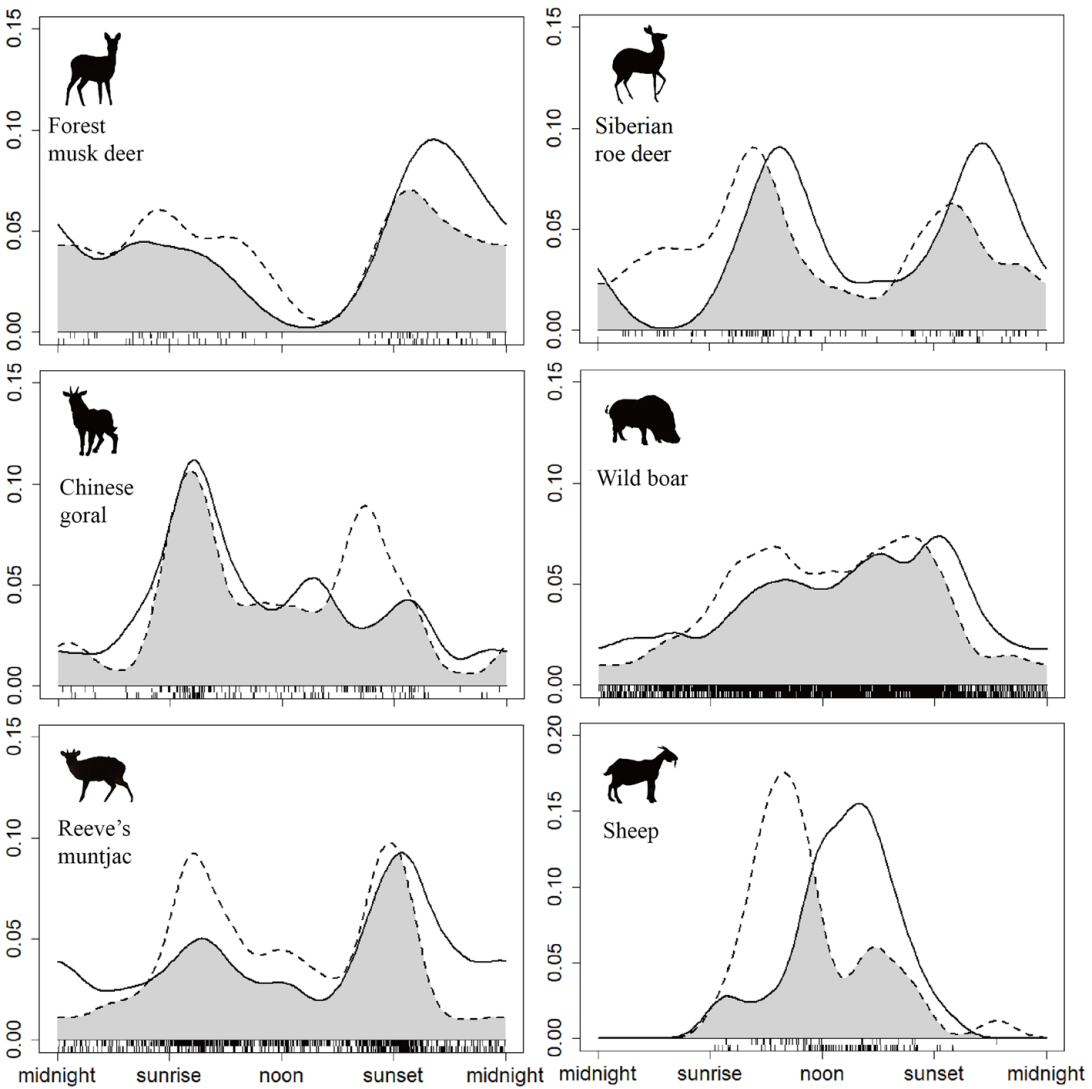
Seasonal variation in activity patterns of wild ungulates and domestic sheep, cold season (solid line) and warm season (dotted line). Grey area under the curves represents the temporal overlap coefficient (∆).

Forest musk deer were primarily active at night in both cold and warm seasons, with peak activity after sunset. And its peak activity was shifted back in time and peak activity frequency was increased during the cold season compared to the warm season (Fig. 2). Chinese gorals and Reeve’s muntjacs were crepuscular animals. In the warm season, both species had two activity peaks at dawn and dusk. While in the cold season, the dusk activity density of Chinese gorals and the dawn activity density of Reeve’s muntjacs reduced, and the two species showed different peaks of activity (Fig. 2). Siberian roe deer exhibited mainly diurnal and crepuscular activity, with bimodal activity observed in both seasons. Activity peaks occurred after sunrise and after sunset in the cold season, shifting to sunset in the warm season (Fig. 2). Wild boars were cathemeral and active throughout the day, with more activity observed during the day than at night. Compared to the warm season, they reduced activity in the morning and increased activity after sunset tn the cold season (Fig. 2). In addition, domestic sheep were primarily diurnal, with peak activity in the morning during the warm season and in the afternoon during the cold season (Fig. 2).

### Daily activity overlaps among species

**Figure 3:**
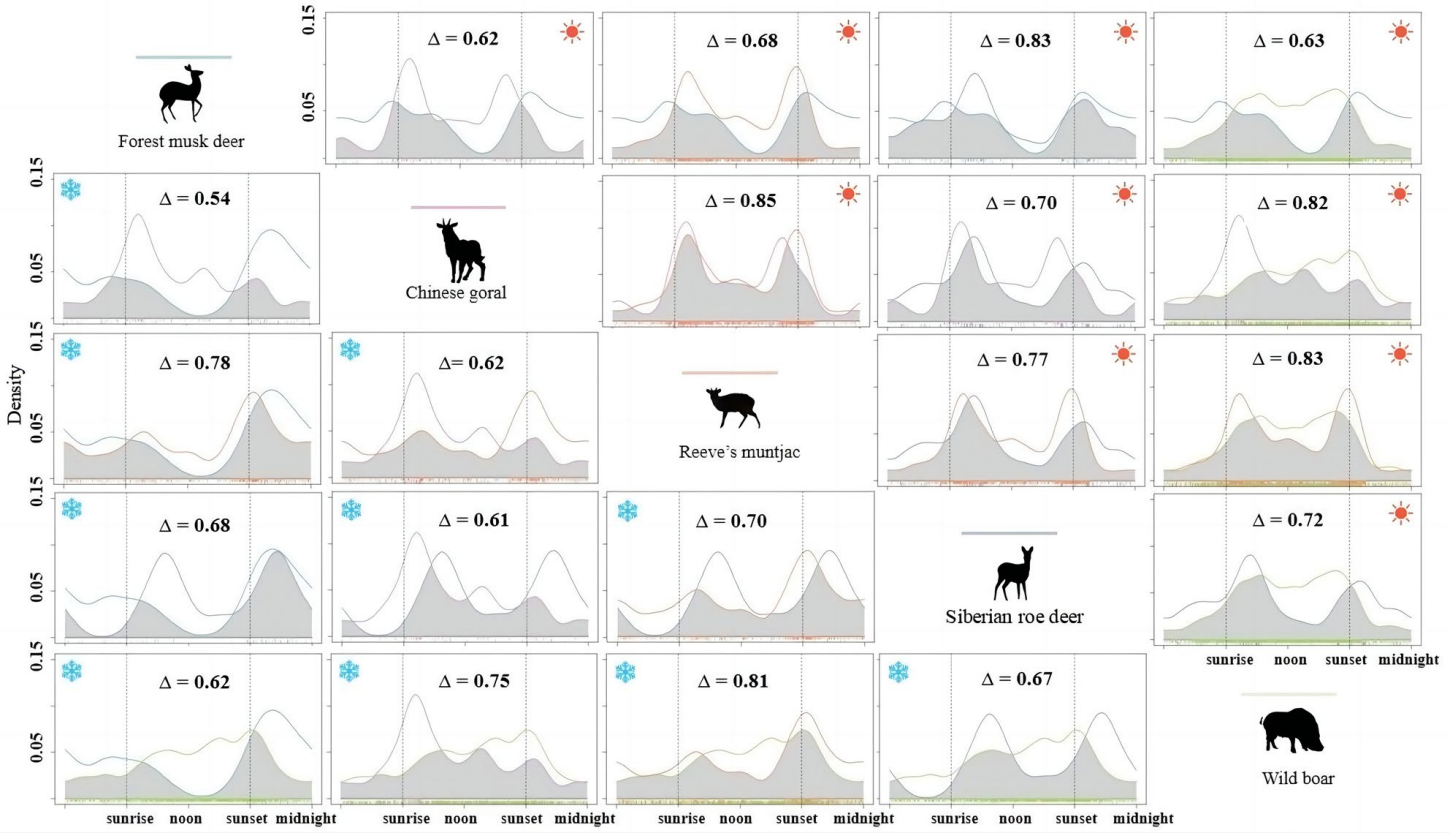
Comparisons between the daily activity patterns of five species of ungulates in cold seasons (marked with blue snowflake symbols on the left) and warm seasons (marked with red sun symbols on the right). The vertical dashed lines representing sunrise and sunset times. Grey area under the curves represents the temporal overlap coefficient (∆).

We calculated temporal overlap coefficients between pairs of wild ungulate species in the cold and warm seasons, respectively, to explore temporal niche partitioning among ungulates (Table 2, Fig. 3). During the cold season, the overlap degree between Reeve’s muntjac and wild boar was highest, with an overlap coefficient of 0.81. The temporal overlap coefficient of forest musk deer and Chinese goral was the lowest, at just 0.54 (*P* < 0.01) (Table 2, Fig. 3). In the warm season, the activity peaks of Chinese goral and Reeve’s muntjac were highly overlapped, with the highest overlap coefficient of 0.85 (Table 2, Fig. 3). The overlap level between forest musk deer and Chinese goral was still the lowest, with an overlap coefficient of 0.62 (*P* < 0.01) (Table 2, Fig. 3). Except for the pair of forest musk deer and Chinese goral, the interspecific temporal overlap between pairs of wild ungulates was higher in the warm season than in the cold season.

**Table 2:** Interspecific overlap coefficients of five wild ungulates and domestic sheep in the cold and warm seasons estimated based on kernel density estimates.

Species pairs	Cold season			Warm season		
	Overlap coefficient	CI	P value	Overlap coefficient	CI	P value
Wild–domestic						
Sheep-Forest musk deer	0.25	0.15–0.36	< 0.01	0.37	0.22–0.52	< 0.01
Sheep-Chinese goral	0.46	0.34–0.59	< 0.01	0.54	0.39–0.59	< 0.01
Sheep-Reeve’s muntjac	0.40	0.30–0.49	< 0.01	0.54	0.42–0.65	< 0.01
Sheep-Roe deer	0.43	0.25–0.61	< 0.01	0.51	0.38–0.64	< 0.01
Sheep-Wild boar	0.57	0.49–0.66	< 0.01	0.64	0.50–0.77	< 0.01
Wild–wild						
Forest musk deer-Chinese goral	0.54	0.42–0.65	< 0.01	0.62	0.49–0.76	< 0.01
Forest musk deer-Reeve’s muntjac	0.78	0.68–0.88	0.02	0.68	0.57–0.79	< 0.01
Forest musk deer-Siberian roe deer	0.68	0.57–0.79	0.10	0.83	0.70–0.94	0.34
Forest musk deer-Wild boar	0.62	0.51–0.72	< 0.01	0.63	0.50–0.74	< 0.01
Chinese goral-Reeve’s muntjac	0.62	0.55–0.76	< 0.01	0.85	0.78–0.92	0.11
Chinese goral-Siberian roe deer	0.61	0.42–0.78	0.02	0.70	0.61–0.80	< 0.01
Chinese goral-Wild boar	0.75	0.65–0.85	< 0.01	0.82	0.73–0.89	0.02
Reeve’s muntjac-Siberian roe deer	0.70	0.58–0.80	0.11	0.77	0.69–0.85	< 0.01
Reeve’s muntjac-Wild boar	0.81	0.68–0.91	< 0.01	0.83	0.79–0.88	< 0.01
Siberian roe deer-Wild boar	0.67	0.47–0.85	0.27	0.72	0.65–0.80	< 0.01

### Daily activity comparison with livestock

**Figure 4:**
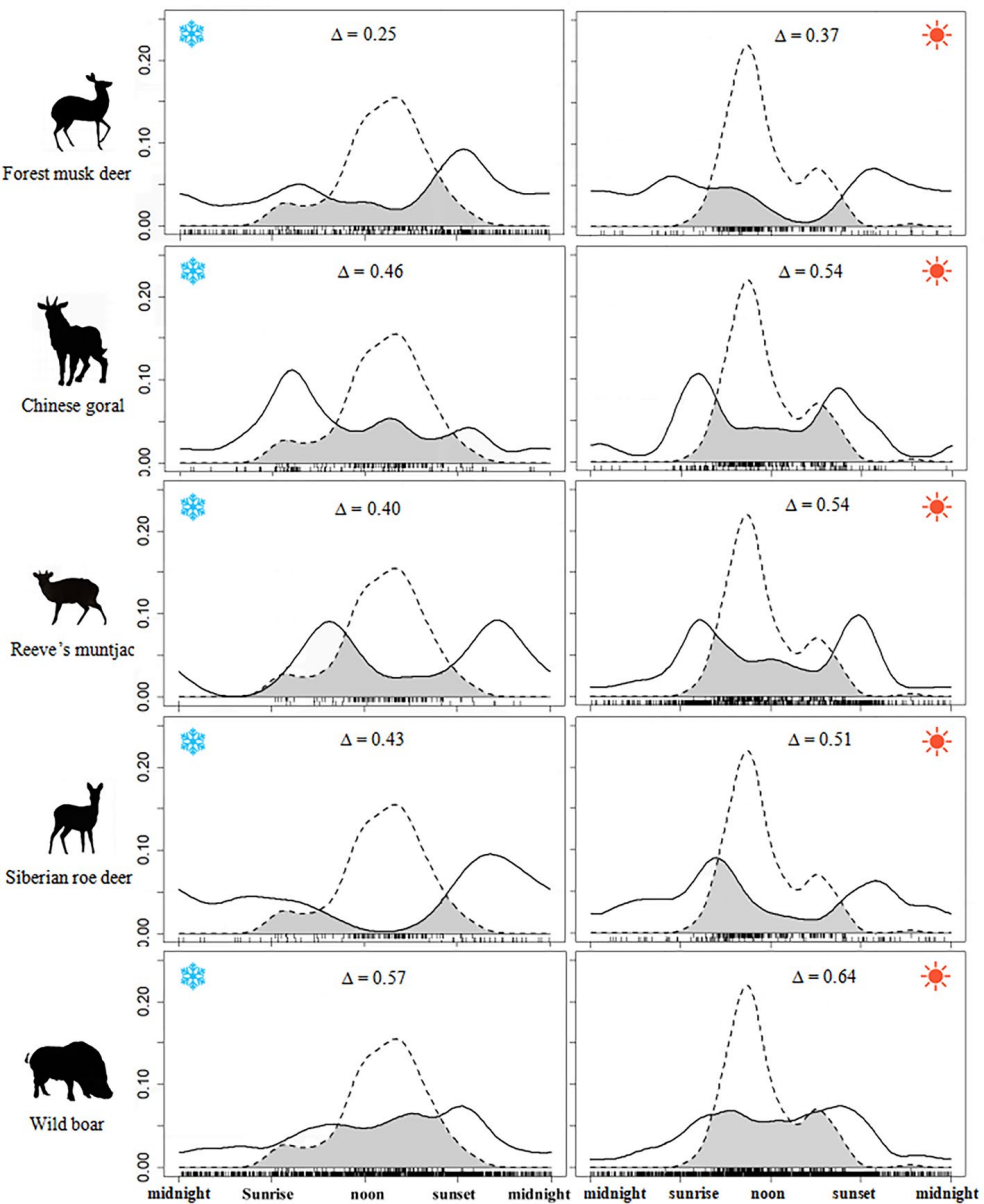
Schematic diagram of the overlapping daily activity rhythms of the five wild ungulates and domestic sheep in the cold season (marked with blue snowflake symbols on the left) and warm season (marked with red sun symbols on the right). Grey area under the curves represents the temporal overlap coefficient (∆).

Our results showed that activity patterns of all five wild ungulates differed significantly from those of domestic sheep, both in the cold and warm seasons (*p* < 0.01) (Table 2, Fig. 4). With the exception of wild boar, ungulates had the lower temporal overlap with domestic sheep than that with any other wild competing species (Table 2). And all wild ungulates avoided peak hours of domestic sheep activity. In both the cold and warm seasons, the highest temporal overlap coefficients with domestic sheep for all wild ungulate species were for wild boar and the lowest for forest musk deer. The temporal overlap coefficients between all wild ungulates and domestic sheep were lower in the cold season than in the warm season (Table 2, Fig. 4).

## Discussion

Based on a 26-month large-scale survey facilitated by camera-trapping technology, this study investigated the seasonal variation in daily activity patterns of five sympatric wild ungulates and free-ranging sheep and their potential temporal interactions. The results were consistent with our prediction that there were differences in daily activity patterns among ungulates, and that these differences were greater in the cold season (because of lower temporal overlap among species). In addition, there were significant differences in activity patterns of wild ungulates and domestic sheep, and wild ungulates had the lower temporal overlap with domestic sheep than that with any other wild competing specie. The study suggested that temporal niche partitioning promoted the coexistence of ungulates. This coexistence strategy was more prominent in the cold season possibly due to resource scarcity.

### Seasonal daily activity pattern

Our findings indicate that forest musk deer exhibit significant nocturnal activity and display dawn and dusk activity peaks consistently throughout the year, consistent with previous studies^59,60^. Interestingly, our results show that forest musk deer exhibit higher peak activity after sunset during the cold season than during the warm season. This could be attributed to shorter daylight hours, which necessitate forest musk deer to spend more time searching for high-energy, protein-rich food sources such as leaves and lichens to meet their heightened energy requirements^61^. Daily activity patterns of Chinese gorals and Reeve’s muntjacs during the warm season showed dusk and dawn activity peaks. However, in the cold season, the dusk activity density of Chinese gorals and the dawn activity density of Reeve’s muntjacs reduced. It was also worth noting that there was a separation of peak activity times for both in the cold season. On the one hand, it suggested that these ungulates increase their activity duration (e.g., exhibiting two activity peaks) during favourable periods (such as daylight hours) in the warm season to increase foraging efficiency and store sufficient fat reserves for potential food shortages in the cold season^62,63^. On the other hand, the staggered activity peak of the two and lower overlap coefficients in the cold season also suggested that ungulates may coexist with competing species through temporal ecological niche partitioning in the resource-poor cold season^46^.

Daily activity patterns of Siberian roe deer differed significantly between cold and warm seasons (p < 0.01). The peak activity of Siberian roe deer in the warm season was at sunrise and sunset, while in the cold season was delayed by 1–2 h compared to the warm season. This implies that temperature may be an important factor influencing daily activity patterns, which is in agreement with the conclusion of Banjade et al.^52^ that seasonal variations in the daily activity patterns of Siberian roe deer are influenced by temperature. In addition, as we predicted, we suggested that activity adjustments of Siberian roe deer in the cold-season were conducive to the temporal niche differentiation from Chinese gorals and Reeve’s muntjacs, thereby reducing interspecific competition in unfavourable environments. Previous studies showed that wild boars exhibited a cathemeral activity pattern without significant activity peaks^64^, and our results provided supportive evidence. According to the seasonal variation of the diet of wild boars^44,65^, the possible explanation of seasonal variations of the daily activity patterns in wild boars may be due to food availability.

### Temporal niche separation among species

The abundance of herbivore species and the absence of top predators in the Baotianman Mountain resulted in more intense competition among ungulate species. We found that five wild ungulates had moderate temporal overlap in the reserve. Among them, forest musk deer is the only nocturnal ungulate in the area, and there are significant differences in body size and feeding habits with other ungulates. Thus, there is likely to be relatively low competition between forest musk deer and other sympatric species. Competition among Chinese goral, Reeve’s muntjac, and Siberian roe deer seems to be of most concern, because they had similar dietary structures^42,66^, had a high overlap of occurrence sites (especially Chinese goral and Reeve’s muntjac, Fig. 1), and all exhibited crepuscular activity with a medium to high temporal overlap. It was worth noting that there were some differences in their activity peaks so temporal niche partitioning could be beneficial in reducing their interspecific competition^11,16,59^. Considering that wild boars are the only omnivorous ungulates in the area and that their body type is larger than that of other ungulates, they have a clear advantage in both resource availability and habitat utilization. From the activity curves, it can be seen that wild boar have significantly different activity patterns (cathemeral activity pattern) from other ungulates, and this temporal scale divergence maybe reduce competition and thus facilitate species coexistence^67–69^. However, the results showed higher temporal overlap coefficients between wild boar and other ungulates, which suggested that we may need to pay more attention to the population dynamics of the wild boar population in the reserve in order to avoid its overpopulation and spread (Supplementary Table S1) and adversely affecting the other ungulates.

In addition, we found that the temporal overlap coefficients in the warm season were higher for all species pairs than in the cold season, except for pair forest musk deer and Reeve's muntjac, partially consistent with our prediction 2. The decrease in temperature and precipitation during the winter month in Baotianman Mountain leads to a decrease in forage and food resources, while low level of resource availability might lead in all ungulate species to more similar patterns of habitat selection and therefore increase temporal avoidance of competition. Thus, temporal niche differentiation may be a strategy to mitigate competition for limited resources in the cold season. The strategy was similar to the study by Hu et al.^46^ on Reeve’s muntjac and black muntjac, which increased differences in daily activity rhythms to coexist in winter. Noted that in our study system, the temporal overlap between most ungulates pairs in the cold season was lower than that in the warm season, but the difference was not significant. This may be an error in temporal overlap evaluation caused by uneven independent events, and larger and more uniform sample sizes may reveal the larger true temporal overlap^70^. However, both seasons showed moderate assessed overlap in all species pairs, suggesting that competition among ungulates in Baotianman Mountain may be more intense than we predicted, and may be an effect of competitive exclusion from free-range sheep.

### Impact of livestock

We found that although all sheep grazing occurred during the day, there was also a significant difference between the peak activity of domestic sheep in the warm and cold seasons. The peak activity of sheep in the warm season was in the morning, while in the cold season, it was in the afternoon. However, the overlap between domestic sheep and wild ungulates was generally smaller than the temporal overlap between wild ungulates in both warm and cold seasons (Table 2), suggesting a potential negative impact of grazing on wild ungulates^71–73^. In the presence of domestic sheep, wild ungulates avoided areas and times with heavy grazing by livestock, especially diurnal animals such as wild boar. Pudyatmoko^35^ also confirmed that in the presence of livestock, some ungulates changed their activity patterns to minimize interactions with livestock. The study suggests that temporal isolation may be a feasible strategy for the coexistence of wild ungulates and domestic livestock. However, further in-depth research is needed on the compression of wildlife temporal niches and the long-term ecological consequences of disturbance competition by livestock.

## Conclusions

Overall, our study focused on the daily activity patterns of five wild ungulates and domestic sheep and the temporal niche partitioning among them during the cold and warm seasons in the Baotianman National Nature Reserve. The results demonstrated seasonal differences in the daily activity patterns of five wild ungulates and confirmed that wild ungulates temporal avoid domestic sheep during both warm and cold seasons in the reserve (due to lower overlap coefficients with sheep and significant differences in activity patterns with sheep), and this temporal ecological niche differentiation potentially promote sympatric coexistence. In addition, the temporal overlap coefficients in the cold season were lower for all species pairs than which in the warm season, except for pair forest musk deer and Reeve's muntjac, indicating that in the resource-poor cold season with limited forage and limited suitable habitats for ungulates, temporal ecological niche partitioning maybe a feasible strategy for promoting the coexistence of ungulates. The study refines the baseline information of these species in Baotianman Mountains, emphasizes the importance of temporal niche partitioning for the sympatric coexistence of ungulates, which is conducive to the in-depth understanding the driving mechanism of activity patterns as well as the coexistence mechanism of ungulates, providing scientific basis wild ungulates conservation and for livestock management strategies.

### Supplementary Information

Supplementary Tables.

## Data Availability

The data that support the findings of this study are available from Wildlife diversity monitoring image data management system (http://www.gscloud.cn). but restrictions apply to the availability of these data, which were used under license for the current study, and so are not publicly available. Data are however available from the authors upon reasonable request and with permission of Wildlife diversity monitoring image data management system (http://www.gscloud.cn).
