# Supplementary material for: Temporal niche partitioning among sympatric wild and domestic ungulates between warm and cold seasons
Source: Sci Rep. 2024 May 8;14:10570. doi: 10.1038/s41598-024-61463-y (PMC11079061; doi:10.1038/s41598-024-61463-y)
Supplement: Supplementary file 1 — Supplementary Tables. [file 41598_2024_61463_MOESM1_ESM.docx]

**Supplementary Table S1** Detection of wild ungulates and human-associated species by camera-trapping in Baotianman National Nature Reserve, Neixiang, from April 2015-June 2017. RAI: Number of independent events per 100 camera days, SO: Percentage of all camera sites.

| Species | Independent events | Warm | | Cold | |
| --- | --- | --- | --- | --- | --- |
|  |  | RAI | SO | RAI | SO |
| Wild ungulates |  |  |  |  |  |
| Forest musk deer  (*Moschus berezovskii*) | 87 | 0.26 | 31.7% | 0.33 | 30.6% |
| Chinese goral  (*Naemorhedus* *griseus*) | 162 | 0.56 | 58.1% | 0.54 | 43.5% |
| Reeve's muntjac  (*Muntiacus reevesi*) | 794 | 3.37 | 62.9% | 1.93 | 58.1% |
| Siberian roe deer  (*Capreolus capreolus*) | 106 | 0.56 | 16.1% | 0.12 | 11.3% |
| Wild boar  (*Sus* *scrofa*) | 2588 | 9.64 | 93.5% | 7.99 | 90.3% |
| Human-associated species |  |  |  |  |  |
| Domestic sheep  (*Ovis aries*) | 123 | 0.22 | 14.5% | 0.66 | 17.7% |
| Domestic cattle  (*Bos taurus*) | 6 | Not analyzed because of low abundance | | | |
| Domestic cat  (*Felis catus*) | 6 | Not analyzed because of low abundance | | | |
| Domestic dog  (*Canis lupus familiaris*) | 25 | Not analyzed because of low abundance | | | |
| Rangers and Visitors  (*Homo sapiens*) | 251 | Not analyzed because of not competing at the same trophic level | | | |

**Supplementary Table S2** Camera-trapping Specifications of Bestguarder SG-990V, Nighthawk

| Specifications | Bestguarder SG-990V, Nighthawk |
| --- | --- |
| Sensor | CMOS Sensor |
| Lens | Auto Control Day/Night Optical Infrared Lens, F=3.1; FOV=62º; PIR=65º |
| Image Color | Daytime Color, Nighttime B/W (Motion freeze technology, which automatically adjusts to night vision at night) |
| Flash | 850 nm LED, low glow |
| PIR Sensing Speed | 0.6-1 sec. |
| Interval time | 0-600 sec. |
| Continuous Shooting | 1-9 shots selectable |
| Shooting range | 0-25 m |
| PIR Sensing Distance | 0-5 m |
| Operating Temperature | -30-60 ºC |
| Operating Humidity | 5-90% |
| Waterproof value | IP66 |
